# Optical properties of epitaxial BiFeO_3_ thin film grown on SrRuO_3_-buffered SrTiO_3_ substrate

**DOI:** 10.1186/1556-276X-9-188

**Published:** 2014-04-23

**Authors:** Ji-Ping Xu, Rong-Jun Zhang, Zhi-Hui Chen, Zi-Yi Wang, Fan Zhang, Xiang Yu, An-Quan Jiang, Yu-Xiang Zheng, Song-You Wang, Liang-Yao Chen

**Affiliations:** 1 Key Laboratory of Micro and Nano Photonic Structures, Ministry of Education, Shanghai Engineering Research Center of Ultra-Precision Optical Manufacturing, Department of Optical Science and Engineering, Fudan University, Shanghai 200433, China; 2 State Key Laboratory of ASIC and System, School of Microeletronics, Fudan University, Shanghai 200433, China

**Keywords:** BiFeO_3_ thin film, Optical properties, Spectroscopic ellipsometry, Lorentz model, Dielectric function, 78.67.-n, 78.20.-e, 07.60.Fs

## Abstract

The BiFeO_3_ (BFO) thin film was deposited by pulsed-laser deposition on SrRuO_3_ (SRO)-buffered (111) SrTiO_3_ (STO) substrate. X-ray diffraction pattern reveals a well-grown epitaxial BFO thin film. Atomic force microscopy study indicates that the BFO film is rather dense with a smooth surface. The ellipsometric spectra of the STO substrate, the SRO buffer layer, and the BFO thin film were measured, respectively, in the photon energy range 1.55 to 5.40 eV. Following the dielectric functions of STO and SRO, the ones of BFO described by the Lorentz model are received by fitting the spectra data to a five-medium optical model consisting of a semi-infinite STO substrate/SRO layer/BFO film/surface roughness/air ambient structure. The thickness and the optical constants of the BFO film are obtained. Then a direct bandgap is calculated at 2.68 eV, which is believed to be influenced by near-bandgap transitions. Compared to BFO films on other substrates, the dependence of the bandgap for the BFO thin film on in-plane compressive strain from epitaxial structure is received. Moreover, the bandgap and the transition revealed by the Lorentz model also provide a ground for the assessment of the bandgap for BFO single crystals.

## Background

BiFeO_3_ (BFO) has attracted extensive research activities as an excellent multiferroic material. It simultaneously exhibits ferroelectricity with Curie temperature (*T*_C_ = 1,103 K) as well as antiferromagnetism with Neel temperature (*T*_N_ = 643 K), and the properties make BFO potential for applications in electronics, data storage, and spintronics [1,2]. Especially, the BFO thin film is paid much attention due to its large spontaneous polarization, which is an order higher than its bulk counterpart [3], and then the BFO thin film combined with nanostructures could be a promising candidate in the above applications [4]. In addition to its structural and electronic properties, optical properties of BFO thin films are focused on [5-9]. However, in the published literatures on optical studies, the BFO thin film is usually directly deposited on perovskite oxide SrTiO_3_ (STO) and DyScO_3_ (DSO) substrate for epitaxial growth. So far, there is no report on optical properties of the BFO thin film with an electrode structure in spite of the fact that the lower electrode is necessary for the study on electronic and ferroelectric properties of the BFO thin film as well as for its applications including nonvolatile memory devices [10]. Since SrRuO_3_ (SRO) is often chosen as the lower electrode for the BFO thin film as well as for the buffer layer to control its nanoscale domain architecture [11], it is desirable to investigate the optical properties of the BFO thin film grown on SRO.

Spectroscopic ellipsometry (SE) is a widely used optical characterization method for materials and related systems at the nanoscale. It is based on the measuring the change in the polarization state of a linearly polarized light reflected from a sample surface which consists of *Ψ*, the amplitude ratio of reflected *p*-polarized light to *s*-polarized light and *Δ*, the phase shift difference between the both [12]. The obtained ellipsometry spectra (*Ψ* and *Δ* at measured wavelength range) are fitted to the optical model for thin film nanostructure, and thus, rich information including surface roughness, film thickness, and optical constants of nanomaterials are revealed [13,14]. Since SE allows various characterizations of the material, our group has studied some thin-film nanostructure using SE methods [15-18].

In this paper, we report the optical properties of epitaxial BFO thin film grown on SRO-buffered STO substrate prepared by pulsed-laser deposition (PLD) and measured by SE. The dielectric functions of STO, SRO, and BFO are extracted from the ellipsometric spectra, respectively. And the optical constants of the BFO thin film are obtained. The bandgap of 2.68 eV for the BFO thin film is also received and is compared to that for BFO thin film deposited on different substrate as well as BFO single crystals.

## Methods

The epitaxial BFO thin film was deposited by PLD on SRO-buffered (111) STO single-crystal substrate. The SRO buffer layer was directly deposited on the STO substrate by PLD in advance. More details about the deposition process can be taken elsewhere [19]. The crystal phases in the as-grown BFO thin film were identified by X-ray diffraction (XRD, Bruker X-ray Diffractometer D8, Madison, WI, USA). The surface morphologies of the BFO thin film were investigated by atomic force microscopy (AFM, Veeco Instruments Inc., Atomic Force Microscope System VT-1000, Plainview, NY, USA). Both XRD and AFM investigation are employed to show growth quality of the BFO thin film for further optical measurement and analysis.

**Figure 1:**
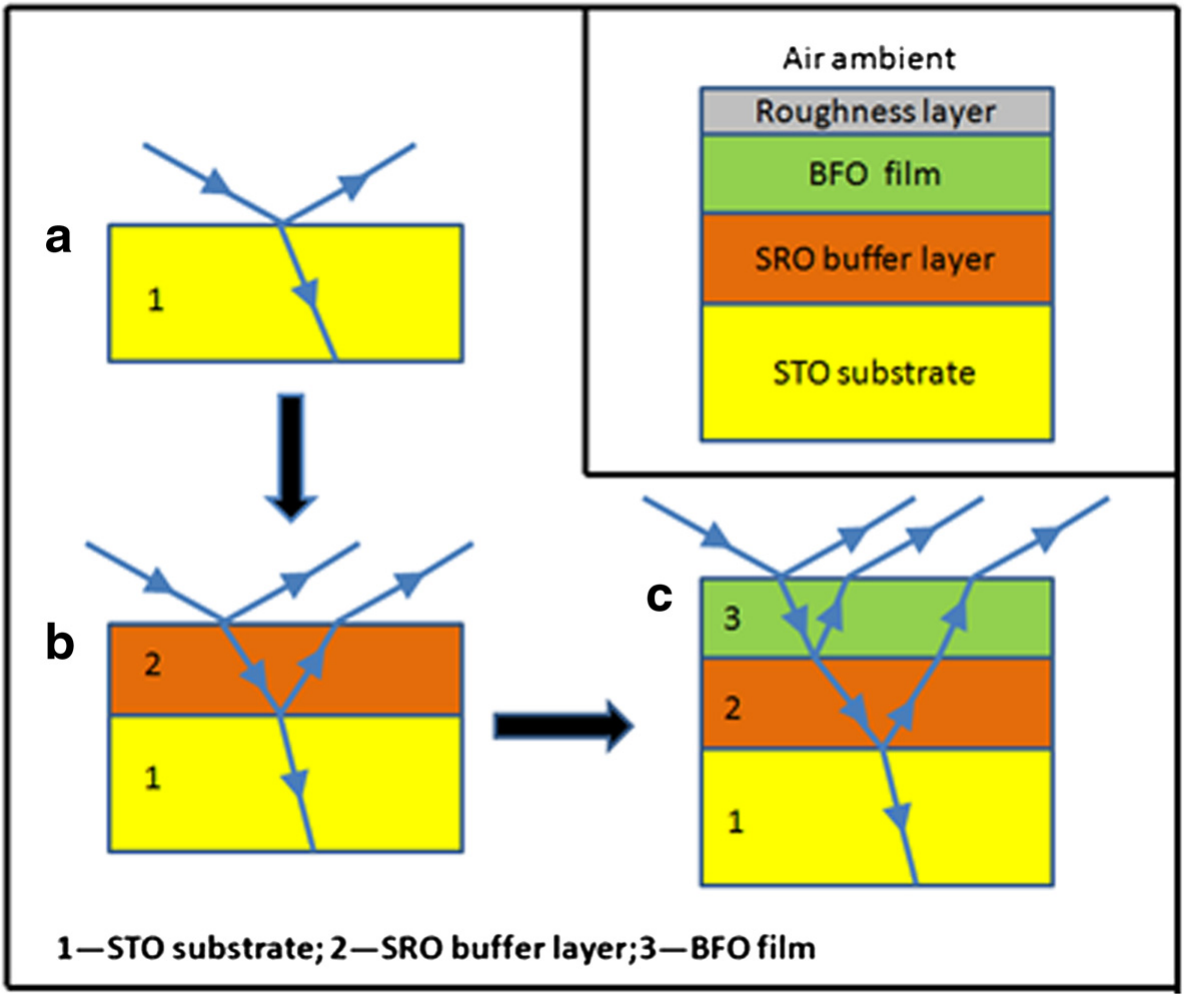
**The schematic of SE measurements on BFO thin film with SRO buffer layer structure. (a)** STO substrate, **(b)** SRO buffer layer, and **(c)** BFO film. The inset is the optical model of the BFO thin film on the SRO-buffered STO substrate.

SE measurements were taken to investigate the optical properties of the BFO film. Considering the optical investigation with respect to a substrate/buffer layer/film structure, we should firstly obtain the optical response of the STO substrate and SRO buffer layer and then research the optical properties of the BFO thin film. The ellipsometric spectra (*Ψ* and *Δ*) were collected for the STO substrate, the SRO buffer layer, and the BFO film, respectively, at an incidence angle of 75° in the photon energy range of 1.55 to 5.40 eV by a SOPRA GES5E spectroscopic ellipsometer (Paris, France), as shown in Figure 1. Afterwards, the ellipsometric data, which are functions of optical constants and layer or film thickness, were fitted to the corresponding optical model depicted in the inset of Figure 1. By varying the parameters of the models in the fitting procedure, the root mean square error (RMSE) is expressed by [17]

(1) $$\mathrm{RMSE}=\sqrt{\frac{1}{2n-m-1}\sum_{i=1}^{n}\left[{\left(\Psi_{i}^{\mathrm{cal}}-\Psi_{i}^{\text{exp}}\right)}^{2}+{\left(\Delta_{i}^{\mathrm{cal}}-\Delta_{i}^{\text{exp}}\right)}^{2}\right]}$$

is minimized. Here, *n* is the number of data points in the spectrums, *m* is the number of variable parameters in the model, and ‘exp’ and ‘cal’ represent the experimental and the calculated data, respectively.

## Results and discussion

**Figure 2:**
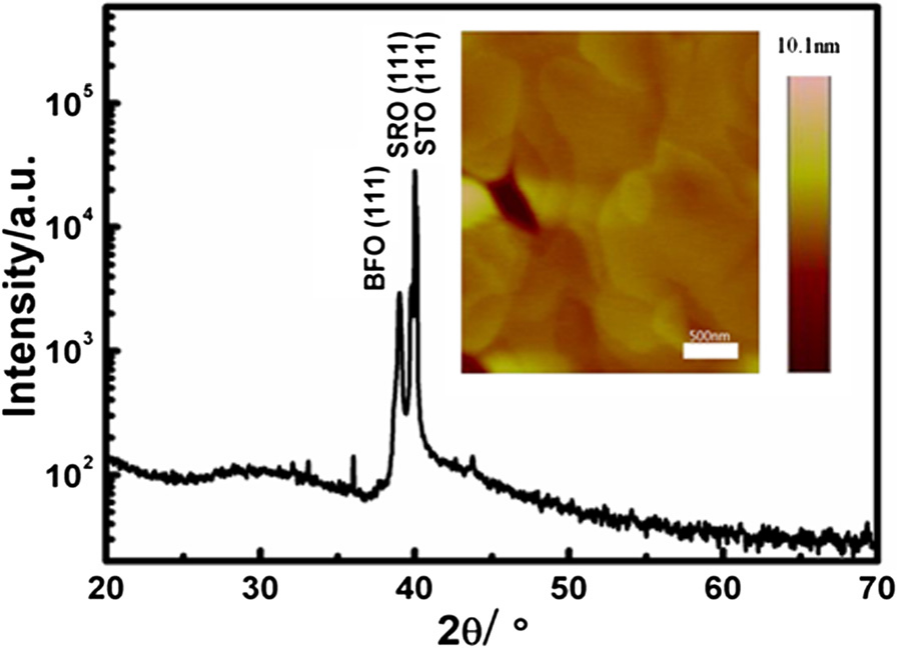
**The XRD pattern of BFO thin film deposited on SRO-buffered STO substrate.** The inset shows its AFM image.

The XRD pattern of the BFO film is displayed in Figure 2 and shows that a strong (111) peak of the BFO matches the closely spaced (111) ones of the SRO and STO, which demonstrates a well-heteroepitaxial-grown film that contains a single phase. As given in the inset of Figure 2, the epitaxial thin film deposited on the SRO/STO substrate is rather dense with *R*q roughness of 0.71 nm. The XRD and AFM results together reveal a smooth epitaxial BFO thin film which is beneficial for the optical measurements.

**Figure 3:**
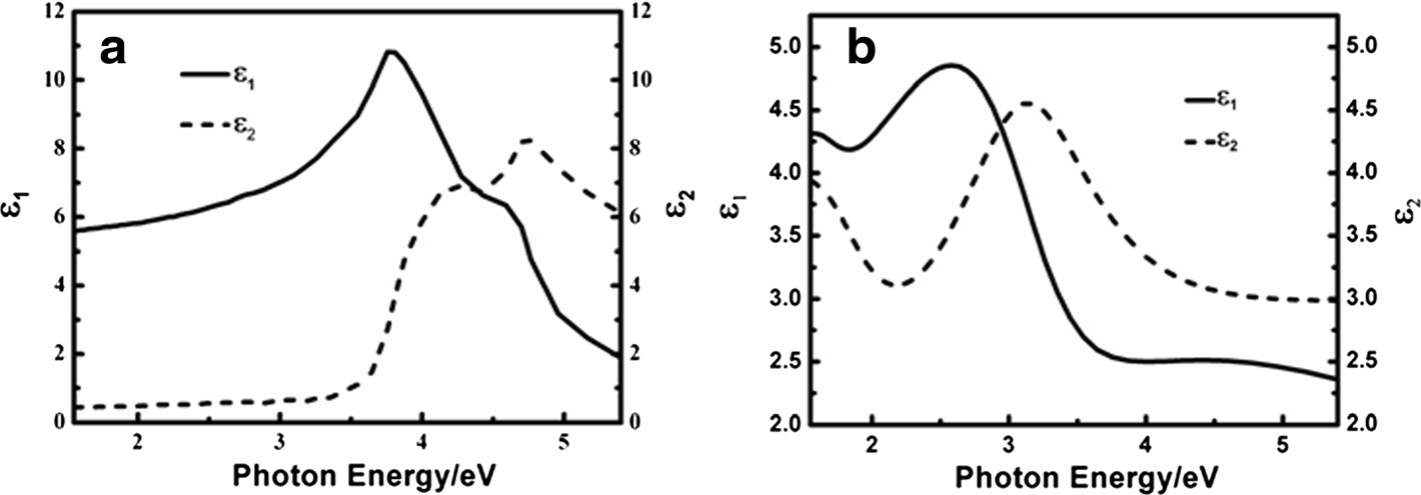
**The dielectric functions for the STO substrate and SRO buffer layer. (a)** STO substrate and **(b)** SRO buffer layer.

The optical response of the STO substrate is calculated by the pseudo-dielectric function [20], and the obtained dielectric functions are shown in Figure 3a, which agrees well with the published literature [21]. The dielectric functions of SRO were extracted by minimizing the RMSE value to fit the ellipsometric data of the SRO buffer layer to a three-medium optical model consisting of a semi-infinite STO substrate/SRO film/air ambient structure. With the dielectric functions calculated for the substrate, the free parameters correspond to the SRO-layer thicknesses and a parameterization of its dielectric functions. The SRO dielectric functions are described in the Lorentz model expressed by [22].

(2) $$\tilde{\epsilon }=\epsilon_{\infty }\left(1+\sum_{j=1}^{4}\frac{A_{j}^{2}}{{\left(E_{\mathrm{center}}\right)}_{j}^{2}-E\left(E-i\nu_{j}\right)}\right)$$

The model parameterization consists of four Lorentz oscillators sharing a high-frequency lattice dielectric constant (*ϵ*_∞_). The parameters corresponding to each oscillator include oscillator center energy *E*_center_, oscillator amplitude *A*_*j*_ (eV) and broadening parameter *ν*_*j*_ (eV). This model yields thickness 105.15 nm for the SRO layer and the dielectric spectra displayed in Figure 3b. The center energy of the four oscillators is 0.95, 1.71, 3.18, and 9.89 eV, respectively, and is comparable to the reported optical transition for SRO at 1.0, 1.7, 3.0, and 10.0 eV [23,24], which indicates that the extracted dielectric functions are reliable.

**Figure 4:**
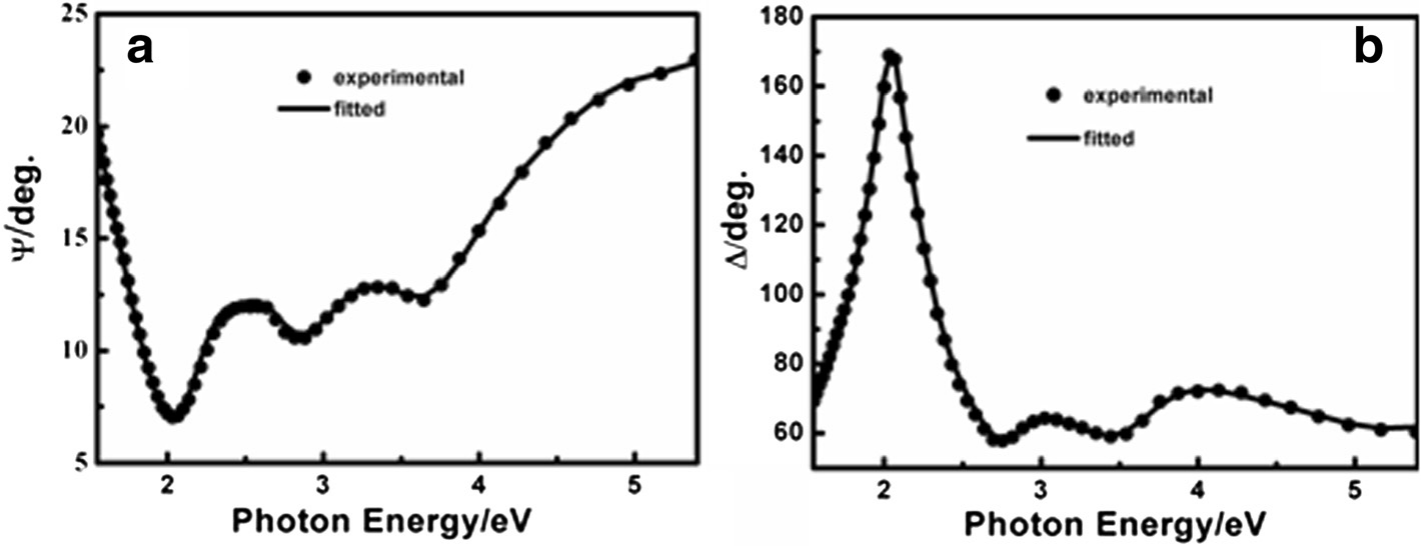
**The measured and fitted ellipsometric spectra for the BFO film. (a)***Ψ* and **(b)** Δ.

The inset of Figure 1 sketches a five-medium optical model consisting of a semi-infinite STO substrate/SRO layer/BFO film/surface roughness/air ambient structure employed to investigate the BFO thin film where the roughness layer is employed to simulate the effect of surface roughness of the BFO film on SE measurement. Since the dielectric functions for the STO substrate and the SRO buffer layer as well as the thickness of SRO layer have been obtained, the free parameters correspond to the BFO film and surface roughness thicknesses and a parameterization of the BFO dielectric functions. The BFO dielectric functions are described by the same four-oscillator Lorentz model as the SRO layer. And the surface roughness layer is modeled on a Bruggeman effective medium approximation mixed by 50% BFO and 50% voids [25]. The fitted ellipsometric spectra (*Ψ* and *Δ*) with RMSE value of 0.26 show a good agreement with the measured ones, as presented in Figure 4. A BFO film of 99.19 nm and a roughness layer of 0.71 nm are yielded by fitting the ellipsometric data to the optical response from the above five-medium model. The roughness layer thickness is exactly consistent with the *R*q roughness from the AFM measurement.

**Figure 5:**
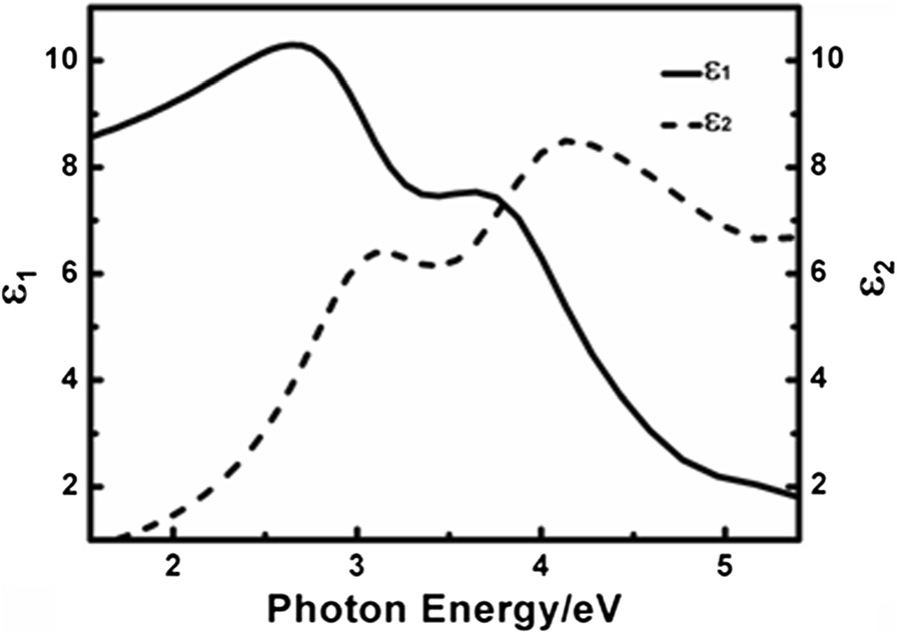
The real and imaginary parts of the dielectric function of the BFO thin film.

The obtained dielectric functions of the BFO thin film are given in Figure 5. In the Lorentz model describing the dielectric functions, the center energy of four oscillators are 3.08, 4.05, 4.61, and 5.95 eV, respectively, which matches well with the 3.09, 4.12, 4.45, and 6.03 eV reported from the first-principles calculation study on BFO [26]. The smallest oscillator energy 3.08 eV is explained either from the occupied O 2*p* to unoccupied Fe 3*d* states or the *d*-*d* transition between Fe 3*d* valence and conduction bands while the other energies can be attributed to transitions from O 2*p* valance band to Fe *3*d or Bi 6*p* high-energy conduction bands [26]. The optical constants refractive index *n* and extinction coefficient *k* are calculated through [27]

**Figure 6:**
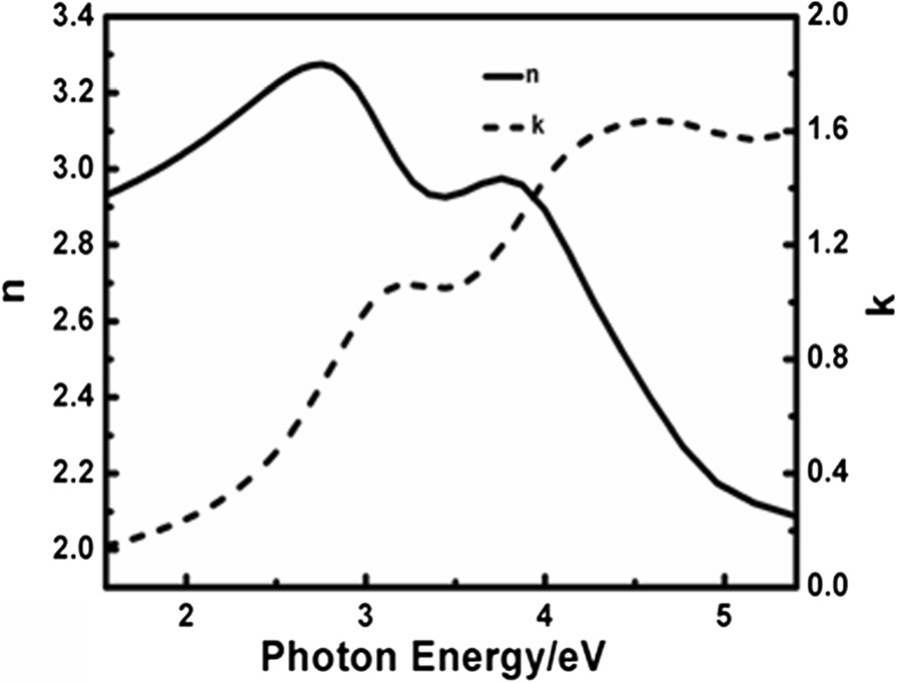
**Refractive index *n *** **and extinction coefficient *k *** **of the BFO film.**

(3) n=ϵ1+ϵ12+ϵ2212/212

(4) k=−ϵ1+ϵ12+ϵ2212/212

and shown in Figure 6.

**Figure 7:**
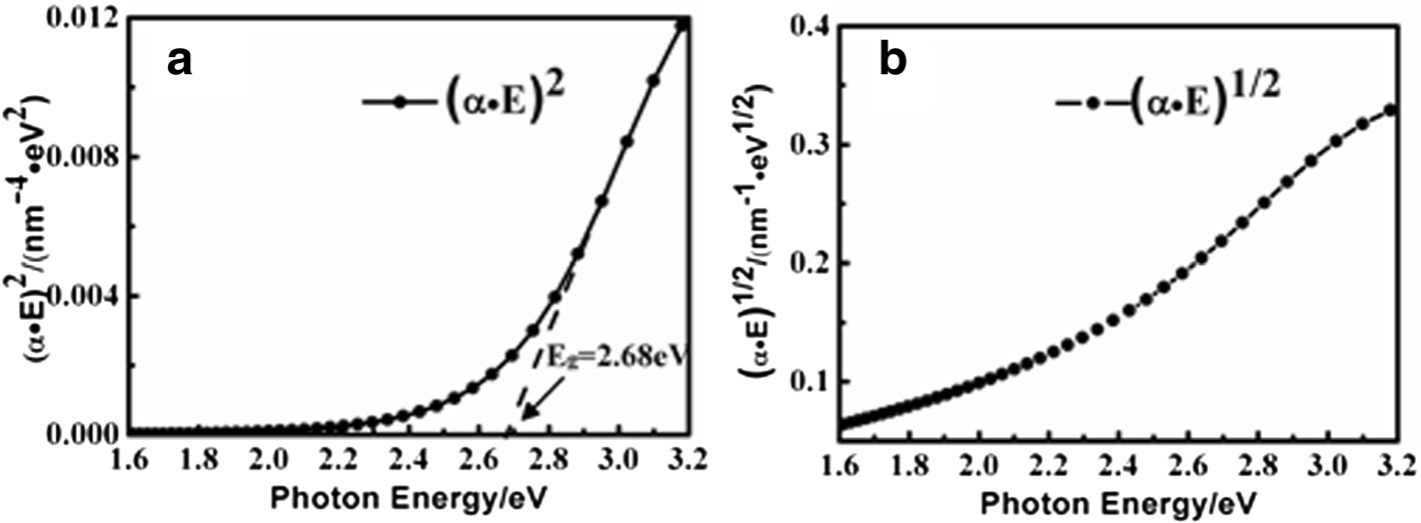
**Plot of (*α***▪***E*)**^***n***^**vs photon energy *E*. (a)***n* = 2 and **(b)***n* = 1/2. The plots suggest that the BFO has a direct bandgap of 2.68 eV.

Plotting (*α*▪*E*)^2^ vs *E* where *α* is the absorption coefficient (*α* = 4π*k*/*λ*) and *E* is the photon energy, a linear extrapolation to (*α*▪*E*)^2^ = 0 at the BFO absorption edge indicates a direct gap of 2.68 eV according to Tauc's principle, as shown in Figure 7a. In the plot of (*α*▪*E*)^1/2^ vs *E* displayed in Figure 7b, no typical indirect transitions are observed in the spectra range [28], suggesting that BFO has a direct bandgap. The bandgap 2.68 eV obtained from the Lorentz model to describe dielectric functions of the BFO thin film is less than the reported 2.80 eV from the Tauc-Lorentz (TL) model [6]. Since the TL model only includes interband transitions [29], intraband transitions and defect absorption taken account into the Lorentz model could impact the received bandgap. In addition, it is reported that there is photoluminescence emission peak at 2.65 eV for the BFO film ascribed to Bi^3+^-related emission [30]. Thus, it is reasonable to believe that the near-band-edge transition contributes to our shrunk bandgap.

On the other hand, it deserves nothing that there is controversy about bandgap sensitivity of the epitaxial thin film to compressive strain from heteroepitaxial structure [5,7]. Considering that the degree of compressive stress imposed by the epitaxial lower layer progressively decreases with increasing BFO thickness [3], our result 2.68 eV from the BFO thin film prepared by PLD with a 99.19-nm thickness is compared to the reported ones of the BFO film on DSO or STO with comparable thickness as well as that deposited by PLD, as listed in Table 1.

**Table 1:** Bandgap of BFO thin film (prepared by PLD) on different substrate

**Bandgap (eV)**	**Substrate**	**Film thickness (nm)**
2.68 (this work)	SRO-buffered STO	99.19
2.67 [8]	DSO	100
2.80 [7]	Nb-doped STO	106.5

The bandgap of BFO on SRO is almost the same as that on DSO and is smaller than that on Nb-doped STO. It is noted that the in-plane (IP) pseudocubic lattice parameter for SRO and DSO is 3.923 and 3.946 Å [11], respectively, while STO has a cubic lattice parameter of 3.905 Å [7]. Considering the IP pseudocubic lattice parameter 3.965 Å for BFO [11], the compressive strain for the BFO thin film deposited on STO substrate is larger than that on SRO and DSO. Thus, the more compressive strain imposed by the heteroepitaxial structure, the larger bandgap for the BFO thin film, which agrees with the past report [7].

The obtained direct bandgap 2.68 eV of the epitaxial BFO thin film is comparable to 2.74 eV reported in BFO nanocrystals [31] but is larger than the reported 2.5 eV for BFO single crystals [32]. This can be understood because even for the epitaxial thin film, the existence of structural defect such as grain boundaries is evitable, which will result in an internal electric field and then widen the bandgap compared to single crystals. On the other hand, a bandgap of 3 eV for BFO single crystals through photoluminescence investigation is also reported [33]. The broad and asymmetric emission peak at 3 eV in the photoluminescence spectra presented in [33] is attributed to the bandgap together with the near-bandgap transitions arising from oxygen vacancies in BFO. However, the Lorentz model employed to depict BFO optical response in our work reveals the existence of a 3.08-eV transition, which is the transition from the occupied O 2*p* to unoccupied Fe 3*d* states or the *d*-*d* transition between Fe 3*d* valence and conduction bands rather than the bandgap [26]. Therefore, the broad and asymmetric peak is more likely to be explained as the overlap of the 3.08-eV transition and the bandgap transition with lower energy.

## Conclusions

In summary, the optical properties of the epitaxial (111) BFO thin film grown on SRO-buffered STO substrate by PLD were investigated. The XRD and AFM analysis indicated that the BFO thin film sample is grown well with epitaxial structure and smooth surface. Then SE measurements were taken to get the ellipsometric spectra of the STO substrate, the SRO buffer layer and the BFO thin film, respectively, in the photon energy range 1.55 to 5.40 eV. The dielectric functions of STO, SRO, and BFO are obtained by fitting their spectra data to different models in which BFO corresponds to a five-medium optical model consisting of a semi-infinite STO substrate/SRO film/BFO film/surface roughness/air ambient structure. The BFO film and surface roughness thickness are identified as 99.19 and 0.71 nm, respectively. The optical constants of the BFO film are determined through the Lorentz model describing the optical response, and a direct bandgap at 2.68 eV is obtained which near-bandgap transitions could contribute to. Moreover, the gap value is compared to the BFO thin film with similar thickness deposited on various substrate prepared by PLD, indicating the dependence of the bandgap for the epitaxial BFO thin film on the in-plane compressive strain. In addition, the transition at 3.08 eV disclosed by the Lorentz model in our work suggests that the bandgap of BFO single crystals is less than 3 eV as previously reported. The results given in this work are helpful in understanding the optical properties of the BFO thin film and developing its application in optical field.

## Abbreviations

BFO: BiFeO_3_; STO: SrTiO_3_; DSO: DyScO_3_; SRO: SrRuO_3_; SE: spectroscopic ellipsometry; PLD: pulsed-laser deposition; XRD: X-ray diffraction; AFM: atomic force microscopy; RMSE: root mean square error; TL: Tauc-Lorentz; IP: in-plane.

## Competing interests

We declare that we have no competing interests.

## Authors' contributions

JPX carried out the optical measurements, analyzed the results, and drafted the manuscript. RJZ proposed the initial work, supervised the sample analysis, and revised the manuscript. ZHC grew the sample. ZYW and FZ performed the XRD and AFM measurements. XY helped dealing with the SE experimental data. AQJ helped the sample growth. YXZ, SYW, and LYC supervised the sample measurements. All authors read and approved the final manuscript.
